# Antivenomics as a tool to improve the neutralizing capacity of the crotalic antivenom: a study with crotamine

**DOI:** 10.1186/s40409-017-0118-7

**Published:** 2017-05-12

**Authors:** Ricardo Teixeira-Araújo, Patrícia Castanheira, Leonora Brazil-Más, Francisco Pontes, Moema Leitão de Araújo, Maria Lucia Machado Alves, Russolina Benedeta Zingali, Carlos Correa-Netto

**Affiliations:** 1 Laboratório de Hemostase e Venenos, Instituto de Bioquímica Médica Leopoldo de Meis, Universidade Federal do Rio de Janeiro (UFRJ), Rio de Janeiro, RJ Brasil; 2 Departamento de Antígenos e Cultivo Celular, Instituto Vital Brazil, Niterói, RJ Brasil; 3 Núcleo Regional de Ofiologia de Porto Alegre (NOPA), Museu de Ciências Naturais, Fundação Zoobotânica do Rio Grande do Sul, Avenida Dr. Salvador França, 1427, Porto Alegre, RS Brasil

**Keywords:** Antivenom production, Antivenomics, *Crotalus durissus*, Crotamine, Crotoxin, Geographic venom variation

## Abstract

**Background:**

Snakebite treatment requires administration of an appropriate antivenom that should contain antibodies capable of neutralizing the venom. To achieve this goal, antivenom production must start from a suitable immunization protocol and proper venom mixtures. In Brazil, antivenom against South American rattlesnake (*Crotalus durissus terrificus*) bites is produced by public institutions based on the guidelines defined by the regulatory agency of the Brazilian Ministry of Health, ANVISA. However, each institution uses its own mixture of rattlesnake venom antigens. Previous works have shown that crotamine, a toxin found in *Crolatus durissus* venom, shows marked individual and populational variation. In addition, serum produced from crotamine-negative venoms fails to recognize this molecule.

**Methods:**

In this work, we used an antivenomics approach to assess the cross-reactivity of crotalic antivenom manufactured by IVB towards crotamine-negative venom and a mixture of crotamine-negative/crotamine-positive venoms.

**Results:**

We show that the venom mixture containing 20% crotamine and 57% crotoxin produced a strong immunogenic response in horses. Antivenom raised against this venom mixture reacted with most venom components including crotamine and crotoxin, in contrast to the antivenom raised against crotamine-negative venom.

**Conclusions:**

These results indicate that venomic databases and antivenomics analysis provide a useful approach for choosing the better venom mixture for antibody production and for the subsequent screening of antivenom cross-reactivity with relevant snake venom components.

## Background

For over a century, antivenoms have remained the only effective treatment for snakebite. An important technical consideration in the production of antivenoms is to use suitable mixtures of venoms (as antigens) in order to produce neutralizing antibodies against the venom of the intended species. Although antivenom administration constitutes an effective therapy against envenomation, the occurrence of inter- and intraspecies venom variability has led to the need for a more robust understanding of venom composition and antivenom efficacy.

The preparation of representative mixtures of venoms from snake species with a broad geographic distribution is not easy, particularly if there is no venomic-based assessment to facilitate the selection of appropriate venom-producing specimens [1, 2]. In Brazil, six subspecies of *Crotalus durissus* are currently recognized (C*. d. dryinas, C. d. marajoensis, C. d. ruruima, C. d. terrificus, C. d. cascavella,* and *C. d. collilineatus*), with each inhabiting distinct ecosystems and displaying a wide geographical distribution [3–5]. All of these subspecies are capable of producing lethal envenomation in humans, since their venoms exhibit systemic neuro- and myotoxic activities.

Envenomation symptoms are often attributed to the presence of crotoxin and crotamine, although marked differences in the concentration of these toxins among venoms have been documented. For example, crotoxin, a neurotoxic phospholipase A_2_ (PLA_2_), is the main toxin of *C. durissus* venom and accounts for 70–90% of its venom proteome [6–10]. On the other hand, significant variation has been observed for crotamine at both individual and population levels, since it accounts from 2 up to 22% of *C. durissus* proteome [9, 11–13]. There is also a positive correlation between the concentration of crotamine present in venom and the level of crotamine gene expression (ranging from 1 to 32 copies per haploid genome) [12].

The Vital Brazil Institute (IVB) is one of three Brazilian institutions that manufacture antivenoms, the others being the Butantan Institute and Ezequiel Dias Foundation (FUNED). Although the crotalic antivenom produced by the three institutions follows the guidelines defined by Brazilian National Health Surveillance Agency (ANVISA), each institution uses its own crotalic antigens. ANVISA has determined that immunization should use crotamine-positive venom obtained from specimens that cover the geographical distribution of *C. durissus* [5]. However, determination of the LD_50_ is the only quality control measure required for the venoms.

The use of antivenomics to evaluate antivenom efficacy was first described in an investigation of the immunoreactivity of the polyvalent antivenom produced by the Costa Rican Clodomiro Picado Institute (ICP) against *Bothriechis lateralis* and *Bothriechis schlegelii* venoms [14]. Subsequently, antivenomics has emerged as a logical extension of venomic studies and has been applied to numerous medically relevant species [1, 15, 16]. In addition, antivenomics protocols have been extensively revised and improved, and used in pre-clinical studies to assess the efficacy of antivenoms and their potential clinical applicability across the geographical range of a species [1, 2, 17–20].

In a previous study, we applied a first generation antivenomics approach to examine the immunoreactivity of crotalic antivenom against subspecies of Brazilian rattlesnakes. The results indicated that the crotalic antivenom was devoid of antibodies capable of recognizing and binding to crotamine [9]. This finding suggested either that the venom used in the production of the crotalic antivenom was devoid of crotamine, or that the low molecular mass of crotamine (4.8 kDa) meant that this cationic polypeptide could be a poor immunogen in horses. In order to explore further this question, the current study applied a second generation of antivenomics approach to examine the cross-reactivity of the crotalic antivenom produced at IVB using a pool of crotamine-negative and crotamine-positive venoms. Our results showed that using the proper immunogenic pool, all components from *Crotalus* venom can be recognized.

## Material and methods

### Venoms and antivenoms

The venoms of *C. d. terrificus* were obtained from captive specimens maintained at the Regional Ophiology Center of Porto Alegre (NOPA) and IVB. Crotamine-positive venom (batch 2014CDU00301) was extracted from 26 adult specimens (10 males and 16 females) housed at NOPA. These snakes were collected primarily in Protásio Alves city, in the southern Brazilian state of Rio Grande do Sul. Crotamine-negative venom (batch 2014CDU00201) was extracted from 44 adult specimens of both genders maintained by IVB. The latter snakes were originally collected near Juiz de Fora in the state of Minas Gerais. Following venom extraction, samples were centrifuged at 1000 g to remove cell debris, lyophilized and stored at − 20 °C.

In accordance with the guidelines of the Brazilian Pharmacopeia [21], and before preparing the mixture of venoms for immunization, the median lethal doses (LD_50_) for the crotamine-positive (batch 2014CDU00301) and crotamine-negative (batch 2014CDU00201) venoms were determined as a quality control. The data available from internal registers of IVB indicated an LD_50_ of 153 μg/kg, accessed via intraperitoneal (i.p.) route, for the crotamine-positive venom (batch 2014CDU00301) and an LD_50_ of 73 μg/kg, i.p., for the crotamine-negative venom (batch 2014CDU00201). The mixture of venoms for immunization was obtained by combining equal amounts of crotamine-positive and negative venoms.

The crotalic antivenom used in this study was produced at IVB (batches SAC085204b and SAC155204F), based on the guidelines of Brazilian Pharmacopeia, and the instructions of ANVISA [5, 21]. This antivenom was of equine origin and consisted of purified F(ab’)_2_ fragments. Antivenom SAC085204b, which expired in 2011, was from the same batch used in our previous antivenomics study [9]. The expiry date of the antivenom batch SAC155204F is October, 2018.

### RP-HPLC venom fractionation

Venom composition was assessed by reversed-phase high-performance liquid chromatography (RP-HPLC) using a Shimadzu Prominence HPLC system. Pooled crotamine-positive (batch 2014CDU00301) and pooled crotamine-negative (batch 2014CDU00201) venom samples were resuspended in 200 μL of 0.1% TFA and applied to a Teknokroma Europa C_18_ column equilibrated with solvent A (0.1% trifluoroacetic acid – TFA). Bound proteins were eluted with discontinuous gradient of solvent B (0.1% TFA in 100% of acetonitrile) at a flow rate of 1 mL/min. For RP-HPLC, we used the same gradient conditions applied in the previous proteomic characterization of *C. d. terrificus* [9]. The elution conditions were: isocratic at 5% B for 10 min, followed by a gradient of 5-15% B for 20 min, 15–45% B for 120 min and 45−70% B for 20 min, with a final isocratic step of 70% B for 5 min. The elution profile was monitored at 214 nm in all experiments. Specific toxin families were identified by comparison of the chromatographic profile of each fraction with the RP-HPLC results from previous venomic analyses of *C. d. terrificus* [8, 9].

### Antivenomics

A second-generation antivenomics method was used, as previously described by Pla et al. [17]. Briefly, 1 mL of NHS-activated Sepharose 4 Fast Flow resin (GE Healthcare) was washed with 10–15 mL of 1 mM HCl and then packed into a column. The column was equilibrated with 2 mL of coupling buffer (0.2 M NaHCO3, 0.5 M NaCl, pH 9.3) at pH 7–8. Fifty milligrams of F(ab’)2 fragments purified from crotalic antivenom was then loaded onto the column and incubated for 4 h at room temperature, according to the manufacturer’s instructions. Unbound F(ab’)2 was washed from the column with equilibration buffer, collected and analyzed by SDS-PAGE. The amount of bound F(ab’)2 was determined by quantifying the unbound antibody densitometrically after SDS-PAGE, using a standard curve obtained by loading known amounts of F(ab’)_2_ molecules (1–5 μg) from the original antivenom. The efficiency of coupling (based on densitometric analysis) was >90% for both antivenoms.

After the removal of unbound F(ab’)2, unreacted groups of the resin were blocked by incubation with 1 mL of 0.1 M Tris-HCl, pH 8.0 on an orbital shaker, overnight at 22–25 °C. The columns were subsequently washed alternately with three volumes of 0.1 M acetate buffer/0.5 M NaCl, pH 4–5, and 0.1 M Tris-HCl, pH 8.5. This treatment was repeated six times.

Before incubation with the venoms, the columns were equilibrated with five volumes of phosphate-buffered saline (PBS). For the immunoassay, 300 μg of *C. d. terrificus* venom, representing a venom:antivenom ratio of 1:150, was dissolved in 1 mL of PBS and applied to the column followed by incubation for 4 h at 25 °C on an orbital shaker. After the incubation, the columns were washed five times with PBS and the unbound material was collected. Immunobound proteins were eluted with 5 mL of buffer (0.1 M glycine, pH 2.0), and neutralized with neutralization buffer (1 M Tris-HCI, pH 9.0). Venom proteins from the immunoaffinity column prepared with antivenom SAC085204b were fractionated by RP-HPLC using a Teknokroma Europa C_18_ column on a Shimadzu Prominence HPLC system, whereas venom proteins from the immunoaffinity column prepared with antivenom SAC155204F were fractionated by RP-HPLC using a Shimadzu (10Avp) HPLC system. Proteins were eluted by washing the columns isocratically with 5% B for 5 min, followed by a gradient of 5–25% B for 5 min, 25–45% B for 60 min and 45–70% for 10 min, with a final isocratic step of 70% B for 5 min at a flow rate of 1 mL/min. Protein detection was performed at 214 nm.

## Results

In a previous study, we reported that the antivenom raised against crotamine-negative *C. d. terrificus* venom failed to recognize crotamine in crotamine-positive venom [9]. To investigate the reason for this lack of immunoreactivity, the present study used a second-generation antivenomics protocol to assess the cross-reactivity of a new batch of antivenom (SAC155204F) raised against a mixture of crotamine-positive and crotamine-negative venoms.

**Fig. 1:**
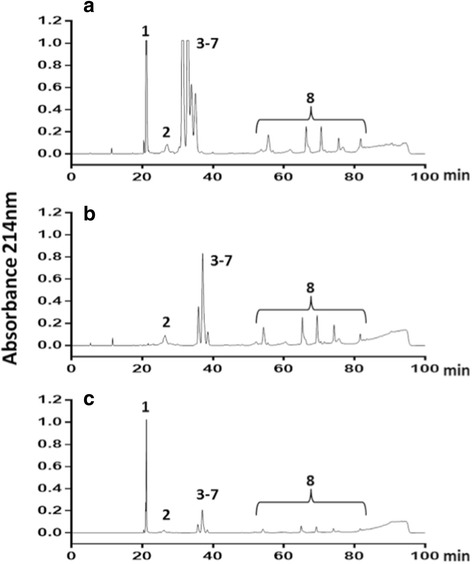
Antivenomics analyses of crotalic antivenom manufactured by IVB. **a** About 300 μg of crotamine-positive venom separated by RP-HPLC using second-generation antivenomics protocols [17]. The elution conditions were: isocratic gradient with 5% of 0.1% TFA in 100% of acetonitrile (solvent B) for 5 min followed by a 5–25% B for 5 min, 25–45% B for 60 min and 45–70% B for 10 min, with a final isocratic step of 70% B for 5 min at a flow rate of 1 mL/min. **b** and **c** the RP-HPLC profiles of retained and non-retained venom toxins on anticrotalic (batch SAC085204b) affinity column, respectively. Protein families associated with HPLC peaks: 1 –crotamine, 2 – disintegrin, 3–7 – crotoxin, and 8 – fractions of low expression toxins including D49-PLA_2_, serine protease (gyroxin), C-type lectin (convulxin) and PIII-metalloproteases

**Fig. 2:**
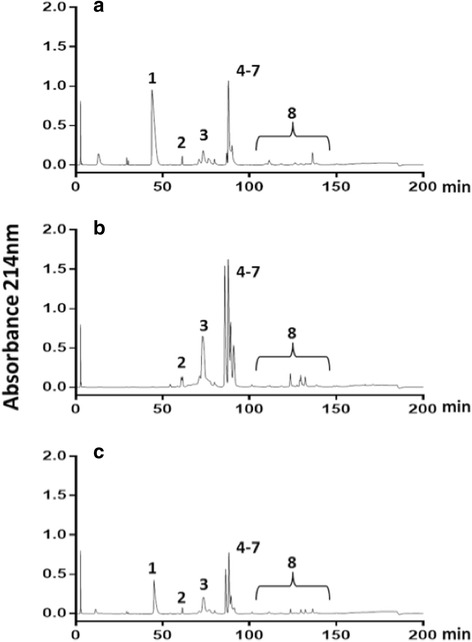
Analyses of *Crotalus durissus terrificus* venoms by RP-HPLC. The venoms of *C. d. terrificus* (batches 2014CDU00301 and 2014CDU00201) were pooled from (**a**) crotamine-positive specimens collected in southern and (**b**) crotamine-negative specimens collected in southeastern Brazil, respectively. The pooled venom for immunization was obtained by combining (**c**) the same ratio of crotamine-positive and crotamine-negative venoms. The elution conditions were: isocratic gradient with 5% of 0.1% TFA in 100% of acetonitrile (solvent B for 10 min followed by 5–15% B for 20 min, 15–45% B for 120 min and 45–70% B for 20 min, with a final isocratic step of 70% B for 5 min at a flow rate of 1 mL/min. Protein families associated with HPLC peaks were: 1 – crotamine, 2 – disintegrin, 3 – crotoxin acid chain, 4 to 7 – crotoxin basic chain, and 8 – fractions of low expression toxins including D49-PLA_2_, serine protease (gyroxin), C-type lectin (convulxin) and PIII-metalloproteases

Initially, we antivenomics to confirm our previous finding regarding the lack of cross-reactivity between SAC085204b and crotamine. Figure 1 confirms the absence of immunoreactivity. To explore further the lack of immunoreactivity, we immunized horses with a mixture containing equal amounts of crotamine-positive and crotamine-negative venoms. Figure 2 shows the profiles of each venom type and the mixture of both. Based on comparison of the elution time of each fraction from RP-HPLC (Fig. 2) with the RP-HPLC results obtained during previous *C.d. terrificus* venomic characterization [8, 9], we identified the HPLC peaks as: 1 – crotamine, 2 – disintegrin, 3 – crotoxin acid chain, 4 to 7 – crotoxin basic chain, and 8 – low expressed toxins including D49-PLA_2_, serine protease (gyroxin), C-type lectin (convulxin) and PIII-metalloproteases.

Each batch of venom was obtained from specimens collected in the same geographical region as the populations of rattlesnakes were previously characterized by our venomics protocols [9]. Crotamine-positive venom (batch 2014CDU00301, LD_50_ = 153 μg/kg) was provided by NOPA and collected from 26 specimens of *C. d. terrificus* captured mainly in the state of Rio Grande do Sul (Fig. 2a). Crotamine-negative venom (batch 2014CDU00201, LD_50_ = 73 μg/kg) was provided by IVB and collected from 44 specimens found in the state of Minas Gerais (Fig. 2b).

**Fig. 3:**
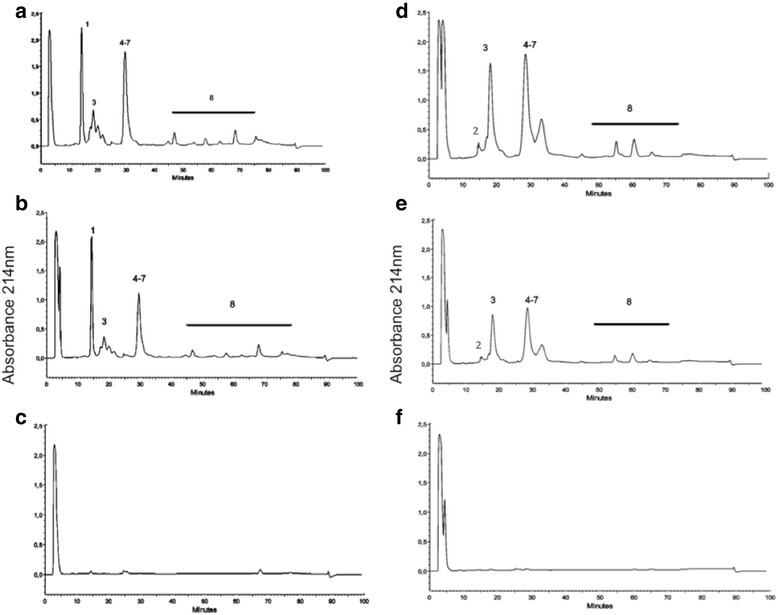
Antivenomics analyses of crotalic antivenom manufactured by IVB from optimized venom mixture. **a** and **d** about 300 μg of crotamine-positive and crotamine-negative venoms separated by RP-HPLC, respectively. **b** and **e** the RP-HPLC profiles of immunocaptured fractions of crotamine-positive and crotamine-negative venom from affinity column with crotalic antivenom SAC155204F (raised against a pool of both types of venoms), respectively. **c** and **f** the flow-through fraction of crotamine-positive and crotamine-negative venoms, respectively. The elution conditions were: isocratic gradient with 5% of 0.1% TFA in 100% of acetonitrile (solvent B) for 5 min, followed by 5-25% B for 5 min, 25–45% B for 60 min, 45–70% B for 10 min, with a final isocratic step of 70% B for 5 min at a flow rate of 1 mL/min. The first peak present in all chromatograms (elution time ~ 3 min) is a technical artifact. Protein families associated with HPLC peaks: 1 – crotamine, 2 – disintegrin, 3 – crotoxin acid chain 4 to 7 – crotoxin basic chain and 8 – fractions of low expression toxins including D49-PLA_2_, serine protease (gyroxin), C-type lectin (convulxin) and PIII-metalloproteases

The pooled venoms used in horse immunization were designed to provide a strong immune response against both crotamine and crotoxin (Fig. 3), and contained approximately 20% and 57% of these toxins, respectively (Fig. 2c). The data of antivenomics analysis clearly demonstrated immunoreactivity towards crotamine, as well as crotoxin, from both venom types (Fig. 3b and e). Immunoreactivity was also observed against toxins that were expressed at lower concentrations and accounted for approximately 6% of the overall protein content of the immunizing pool (Fig. 3c and f, fraction 8). This group of toxins, which includes D49-PLA_2_, serine proteases (gyroxin) and P-III metalloproteases, is conserved in others subspecies of *C. durissus* [8, 9].

## Discussion

Despite significant intraspecific venom variability, the beta-neurotoxin crotoxin is the main lethal component in *C. durissus* venom. Its LD_50_ values calculated in mice are between 60 and 180 μg/kg, depending on the rout of administration (intravenous, subcutaneous etc.). Following crotalic envenomation, crotoxin is responsible for the neurotoxicity and local and systemic myotoxicity that leads to acute nephrotoxicity and renal failure [22–26]. On the other hand, the main effect of crotamine is to induce skeletal muscle spasms via interaction with Na^+^ channels [27, 28]. In animal models, crotamine also induces strong paralysis of the hind limbs [29].

Crotamine has a significantly lower toxicity (LD_50_ 6.8 mg/kg, i.e., two orders of magnitude higher than crotoxin) suggesting that it contributes more to prey immobilization than to lethality. However, crotamine has also been shown to contribute to myotoxic, cytotoxic and hemolytic activities that could potentially contribute to the nephrotoxicity often observed after envenomation by *C. durissus* [22, 26, 28, 30, 31]. Crotalic antivenom would be expected to neutralize crotamine and its activities. A major limitation of antivenom therapy after snakebite is that antivenoms with low neutralizing capacity must be administered at higher doses, with severe cases of envenomation requiring high amounts of antivenom [26]. Regarding the production and quality control of antivenoms, each batch must contain similar amount of IgG-derived molecules capable of neutralizing all of the toxic compounds in the venom of a certain specific species.

The venom LD_50_ values reported herein agree with the crotoxin/crotamine concentration for each type of venom. The high content of crotoxin present in crotamine-negative venom likely explains the low LD_50_ value observed for this venom when compared to the LD_50_ of crotamine-positive venom. Although venom LD_50_ values may be similar, they provide little information about the overall venom composition since venoms often vary in their content of crotamine and other venom compounds (e.g., serine proteases, D49-PLA_2s_, and metalloproteinases).

Although methods such as HPLC, SDS-PAGE and ELISA have been proposed to identify and quantify crotamine [32, 33], the ANVISA guidelines neither require standardization of the crotamine concentration in venom pools, nor do they propose any analytical method for the identification and quantification of this toxin. The current ANVISA regulation recommending the use of crotamine-positive venoms for immunization, without knowledge of the actual concentration of this compound in the venom, is problematic and can lead to fluctuations in antibody concentrations among batches.

The results presented in the current study show that the use of pools of venom with low crotamine content may stimulate a weak immune response for this molecule. In addition, crotamine-negative venoms may contain a greater amount of isoforms of the acidic chain of crotoxin, as well as other low expression toxins (Fig. 2b, fractions 8), including D49-PLA_2_, a myotoxin that accounts for 18% of the venom proteome of the northeastern Brazilian rattlesnake *C. d. cascavella* [9]. Herein, we propose that the standardization of a minimal concentration of toxic compounds, especially crotamine and crotoxin in the *C. d. terrificus* venom pools used for immunization, is a valuable and necessary procedure to ensure quality and reproducibility among batches of crotalic antivenom from each manufacturing institution.

## Conclusions

The results of the present study demonstrate the usefulness of antivenomics analyses for choosing the appropriate *C. d. terrificus* venoms for antivenom production. Our findings also show that by using a mixture containing equal amounts of crotamine-positive and crotamine-negative *C. d. terrificus* venoms, we were able to produce an equine antivenom that successfully recognized crotamine in crotamine-positive venom.

## Abbreviations

ANVISA: National Health Surveillance Agency
FUNED: Ezequiel Dias Foundation
i.p.: Intraperitoneal
ICP: Clodomiro Picado Institute
IVB: Vital Brazil Institute
LD_50_: Median lethal doses
NOPA: Regional Ophiology Center of Porto Alegre
PBS: Phosphate-buffered saline
PLA_2_: Phospholipase A_2_
RP-HPLC: Reversed-phase high-performance liquid chromatography
TFA: Trifluoroacetic acid
